# The endocranium and trophic ecology of *Velociraptor mongoliensis*

**DOI:** 10.1111/joa.13253

**Published:** 2020-07-10

**Authors:** J. Logan King, Justin S. Sipla, Justin A. Georgi, Amy M. Balanoff, James M. Neenan

**Affiliations:** ^1^ School of Earth Sciences University of Bristol Bristol UK; ^2^ Department of Anatomy and Cell Biology University of Iowa Iowa City IA USA; ^3^ Department of Anatomy Midwestern University Glendale AZ USA; ^4^ Division of Paleontology American Museum of Natural History New York NY USA; ^5^ Department of Psychological and Brain Sciences Johns Hopkins University Baltimore MD USA; ^6^ Oxford University Museum of Natural History University of Oxford Oxford UK

**Keywords:** Dinosauria, Dromaeosauridae, endosseous labyrinth, neuroanatomy, sensory anatomy, Theropoda

## Abstract

Neuroanatomical reconstructions of extinct animals have long been recognized as powerful proxies for palaeoecology, yet our understanding of the endocranial anatomy of dromaeosaur theropod dinosaurs is still incomplete. Here, we used X‐ray computed microtomography (µCT) to reconstruct and describe the endocranial anatomy, including the endosseous labyrinth of the inner ear, of the small‐bodied dromaeosaur, *Velociraptor mongoliensis*. The anatomy of the cranial endocast and ear were compared with non‐avian theropods, modern birds, and other extant archosaurs to establish trends in agility, balance, and hearing thresholds in order to reconstruct the trophic ecology of the taxon. Our results indicate that *V. mongoliensis* could detect a wide and high range of sound frequencies (2,368–3,965 Hz), was agile, and could likely track prey items with ease. When viewed in conjunction with fossils that suggest scavenging‐like behaviours in *V. mongoliensis*, a complex trophic ecology that mirrors modern predators becomes apparent. These data suggest that *V. mongoliensis* was an active predator that would likely scavenge depending on the age and health of the individual or during prolonged climatic events such as droughts.

## Graphical abstract

We reconstructed the brain cavity and inner ear of *Velociraptor* to better understand its lifestyle and hunting abilities. We found brain structures that are present in modern birds, indicating an exceptional sense of balance and agility. The inner ear also closely resembles that of flightless birds, and *Velociraptor* had a similar hearing ability to ravens. *Velociraptor* was an agile predator that relied heavily on its sense of hearing to hunt, although it would scavenge if given the opportunity.

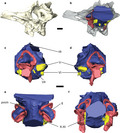

## 1 INTRODUCTION

*Velociraptor mongoliensis* Osborn, 1924 is a velociraptorine dromaeosaur found in Late Cretaceous formations of China and Mongolia (Osborn, 1924; Godefroit *et al*., 2008) that has been made famous in recent years thanks to its portrayal in numerous Hollywood movies. *V. mongoliensis* has also been the subject of a number of cranial and postcranial publications (Sues, 1977; Norell *et al*., 1997; 2004; Barsbold and Osmólska, 1999; Turner *et al*., 2007; Manning *et al*., 2009), with the cranial osteology, including the braincase, being well‐known thanks to the exceptionally preserved specimens found in Mongolia (Barsbold and Osmólska, 1999). Despite this heightened attention, the endocranial anatomy of *V. mongoliensis* has not yet been described. Indeed, the endocranial anatomy of Dromaeosauridae as a whole is still relatively poorly known despite the initial osteological description of *Dromaeosaurus* having occurred almost a century ago (Matthew and Brown, 1922). Since that time, the endocast for *Bambiraptor feinbergi* (Burnham, 2004) has been partially described and portions of the endocast of *Saurornitholestes langstoni*, *V. mongoliensis*,* Tsaagan mangas*, and *Deinonychus antirrhopus* have been measured for quantitative analysis or otherwise imaged (Witmer and Ridgely, 2009; Zelenitsky *et al*., 2011; Balanoff *et al*., 2013). There is, however, a distinct lack of described endocasts with which to expand the palaeobiology of dromaeosaurs in the formal literature to date. Although a few publications have noted and discussed the implications of the large endocranial space in dromaeosaurs (Hopson, 1977; Currie, 1995; Norell *et al*., 2004), relating the endocranial anatomy of velociraptorine dromaeosaurs to their trophic ecology has yet to be done in any capacity.

Evidence for the trophic ecology of *V. mongoliensis*, or at least velociraptorine dromaeosaurs, is provided by a few different sources. The most famous of these, the ‘fighting dinosaurs’ of Inner Mongolia, preserves a glimpse into the predator–prey relationship between *V. mongoliensis* (IGM 100/25) and *Protoceratops andrewsi* (IGM 100/512) (Carpenter, 1998). However, two other *V. mongoliensis* specimens indicate what may be considered scavenging behaviour (Hone *et al*., 2010; 2012). Several previous studies have explored the connection between endocranial anatomy, palaeoecology, and behaviour within theropod dinosaurs. Medium‐ and large‐bodied carnivorous theropods (e.g. tyrannosaurids (Witmer and Ridgely, 2009; Bever *et al*., 2011; Brusatte *et al*., 2016; Kundrát *et al*., 2018; McKeown *et al*., 2020), abelisaurids (Carabajal and Succar, 2015), carcharodontosaurids (Franzosa and Rowe, 2005; Brusatte and Sereno, 2007; Carabajal and Canale, 2010), megaraptorans (Carabajal and Currie, 2017), and allosaurids (Rogers, 1999; Gleich *et al*., 2005)) as well as small and medium‐sized maniraptorans—e.g. oviraptorosaurs (Kundrát, 2007; Balanoff *et al*., 2018), therizinosaurs (Lautenschlager *et al*., 2012), and others (Walsh *et al*., 2009; Zelenitsky *et al*., 2011)—have been the focus of neurosensory studies. These studies often are able to utilize structures reflected in the endocasts such as the olfactory apparatus, cochlear ducts, and optic lobes to reconstruct the posture and sensory capabilities for these extinct taxa. For instance, quantitative and comparative analyses of tyrannosaurids have found that they had the sensory requirements for an active predatory lifestyle (Witmer and Ridgely, 2009), and semicircular canal morphologies have been found to correspond to quadrupedal and bipedal locomotor modes in dinosaurs (Georgi *et al*., 2013). Even in herbivorous theropods, such as *Erlikosaurus*, strong senses of smell, agility, eyesight, and hearing have been estimated (Lautenschlager *et al*., 2012).

With this in mind, we explored the neuroanatomy of *V. mongoliensis* (IGM 100/976) in order to better estimate the trophic ecology and sensory aptitude of this species—thus providing much needed sensory and behavioural data for dromaeosaurs. Here, we describe the anatomy of the hindbrain and inner ear of *V. mongoliensis* using cranial endocasts and compare its neuroanatomy to extant reptilian (including birds) taxa in order to place its sensory abilities into a broad palaeoecological context.

## 2 METHODS

**FIGURE 1:**
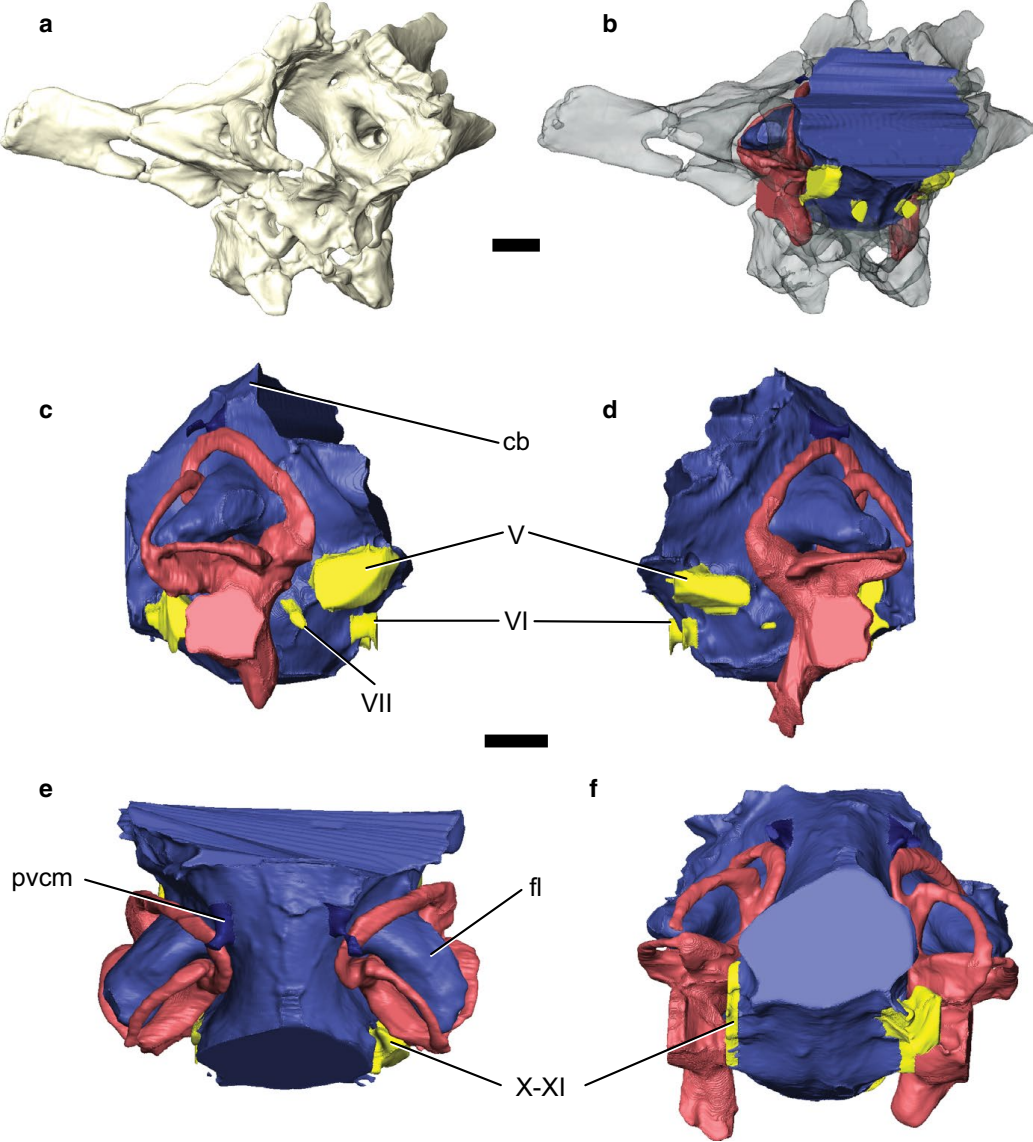
The braincase and endocranium of *Velociraptor mongoliensis* IGM 100/976. (a) Partial braincase in anterolateral view. (b) Braincase rendered transparent, revealing the in situ endocast in anterolateral view. The labelled endocast is presented in the right, (c), and left (d) lateral, dorsal (e), and posterior (f) views. Brain endocast is shown in blue, veins in dark blue, cranial nerves in yellow, and endosseous labyrinth in pink. Scale bars: 5 mm. cb, cerebellum; pvcm, posterior middle cerebral vein; fl, floccular lobes; V, trigeminal nerve; VI, abducens nerve; VII, facial nerve; X–XI, shared foramina for the vagus and accessory nerves

IGM 100/976 was collected as a part of the 1991 Joint Expedition of the Mongolian Academy of Sciences and American Museum of Natural History. This specimen was recovered from the Djadokhta Formation at Tugrugeen Shireh, Mongolia (Norell *et al*., 1997) and consists of a partial skeleton, including an incomplete braincase that is missing the bones anterior to the basisphenoid and supraoccipitals (Figure 1a,b). The braincase is comprised of a few incomplete elements—the exoccipitals, supraoccipital, and basioccipital. These four elements are fused to form an incomplete adult endocranial space where the sutures are obliterated along the surface (Norell *et al*., 2004). Because of its incomplete nature, the endocast preserves the entire hindbrain but only a featureless portion of the midbrain.

IGM 100/976 was scanned at the University of Texas High‐Resolution X‐ray CT Facility in Austin, Texas, USA, producing 1024 × 1024 16‐bit TIFF images. Scan parameters were as follows: 210 kV, 0.11 mA, intensity control on, high‐power mode, no filter, air wedge, no offset, slice thickness 1 line (0.08506 mm), source‐object distance 245 mm, 1,400 views, two samples per view, inter‐slice spacing 1 line (0.08506 mm), field of reconstruction 81 mm (maximum field of view 81.8084 mm), reconstruction offset 8,700, reconstruction scale 4,000. Acquired with 31 slices per rotation and 25 slices per set. Ring‐removal processing based on correction of raw sinogram data using IDL routine ‘RK_SinoRingProcSimul’ with parameter ‘bestof5 = 11’. Reconstructed with beam‐hardening coefficients (0.0, 0.6, 0.1, 0.05), and a rotation of 4 degrees. Total final slices = 450. Segmentation, reconstruction, and measurement collection were conducted in avizo lite (Thermo Fisher Scientific, 9.7.0) and amira 2019.1 (Thermo Fisher Scientific).

The mean and high hearing frequencies for IGM 100/976 were calculated following the method outlined in Walsh *et al*. (2009). To accomplish these reconstructions, we took measurements from the anterior‐most extent of the basisphenoid to the posterior‐most margin of the occipital condyle along with the length of the cochlear duct (Table 1). The two measurements were then used to calculate a cochlear duct‐basisphenoid ratio and then logarithmically transformed. This normalized value was placed into pre‐calculated formulae found in Walsh *et al*. (2009).

**Table 1:** Measurements taken from the endocast of IGM 100/976. Volumes do not account for vascularization, endosseous labyrinths or cranial nerves.

Element measured	
Minimum width	14.44 mm
Maximum width	28.49 mm
Cerebellum height	26.13 mm
Cerebellum width	15.75 mm
Total endocast length	22.79 mm
Pontine flexure angle	132.94°
Floccular lobe length	9.59 mm
Angle of floccular lobe orientation	123°
Total floccular volume	0.40 g/mm^3^
Total volume	5.73 g/mm^3^
Cochlear duct length	11.15 mm
Basisphenoid length	34.71 mm

Institutional abbreviations: IGM—Institute of Geology in Ulaan Baatar, Mongolia; IVPP—Institute of Vertebrate Paleontology and Paleoanthropology, Beijing, China; MPC‐D—Paleontological Laboratory of the Paleontological Center, Ulaan Baatar, Mongolia.

## 3 RESULTS

### 3.1 Cranial endocast

The identifiable regions of the brain preserved in the specimen are limited to the hindbrain: the medulla and cerebellum, including its floccular lobes. The flocculi are situated posterolaterally and orientated posteriorly at 123° (Figure 1c,f) The bodies of the floccular lobes are elongate, roughly circular in cross‐section, and fill most of the space between the anterior and posterior semicircular canals. Each lobe extends well beyond the posterior margin of the anterior canal and almost through the posterior semicircular canal of the endosseous labyrinth. The flocculi together account for approximately 7% of the total hindbrain volume (Table 1).

The medulla is wider than tall and forms an almost oval shape at the foramen magnum. As seen in most other maniraptorans, the medulla is antero‐posteriorly short and narrower than the rest of the hindbrain (Kundrát, 2007; Balanoff *et al*., 2009; Lautenschlager *et al*., 2012) (Table 1). Anteriorly, the medulla exhibits a gentle dorsolateral constriction between it and the cerebellum. Anteriorly, there is a 132.94° angle between the hindbrain and midbrain. This pontine flexure (Table 1) implies that the brain exhibited a gentle curvature and was not all located along the same horizontal plane. This curvature is unsurprising due to its presence in many non‐maniraptoran theropods (Sampson and Witmer, 2007; Witmer and Ridgely, 2009), basal therizinosaurs (Lautenschlager *et al*., 2012), and oviraptorosaurs (Kundrát, 2007; Balanoff *et al*., 2014).

As a whole, few anatomical structures are preserved on the endocast of the cerebellum. The hindbrain lacks a prominent dorsal dural peak overlying the cerebellum that is found in some other maniraptorans such as *Conchoraptor* (Kundrát, 2007) and large‐bodied derived tyrannosaurs (Osborn, 1912; Witmer and Ridgely, 2009; Bever *et al*., 2011; Brusatte *et al*., 2016). The absence of a large dural peak is consistent with another velociraptorine dromaeosaur, *T. mangas* (personal observation by the authors) and basal tyrannosaurs (Kundrát *et al*., 2018); however, it is possible that this portion of the endocast was not preserved.

### 3.2 Cranial nerves and vasculature

Both trigeminal nerves (CN V) are preserved; each exiting the lateral portions of the anteriormost endocast. The trigeminal is preserved as a single nerve that likely diverged into its component branches outside of the braincase as it does in other non‐avian maniraptorans (Figure 1c,d) (Currie, 1995). The abducens nerve (CN VI) is located ventromedial to CN V (Figure 1c,d) and has an anterior trajectory. The endocasts of the abducens nerves are incomplete and project anteriorly only a few millimetres before reaching the anterior limit of the braincase. A short canal for the facial nerve (CN VII) lies on the medulla at the level of the anterior edge of the endosseous labyrinth, just posterolateral to CN V. Both the vagus and accessory nerves (CN X–XI, respectively) exit a single ventrolaterally located foramen along the posterior portion of the braincase (Figure 1e,f). While it is located laterally near the posteriormost part of the braincase, the hypoglossal (CN XII) could not be reliably reconstructed even though the CN XII foramina are visible on the external braincase (Norell *et al*., 2004).

Norell *et al*. (2004) initially described the presence of vasculature along the interior surfaces of the braincase in this specimen of *Velociraptor*, although these could not be reconstructed digitally. The occurrence of small veins in *Velociraptor* would not be surprising considering birds and their close relatives have a thin dural envelope and a majority of their braincase filled with neural tissue (Norell *et al*., 2004; Evans, 2005). Little of the venous architecture is preserved—only the posterior middle cerebral veins are observable along the posterodorsal surface of the cerebellum (Figure 1e).

### 3.3 Endosseous labyrinth

**FIGURE 2:**
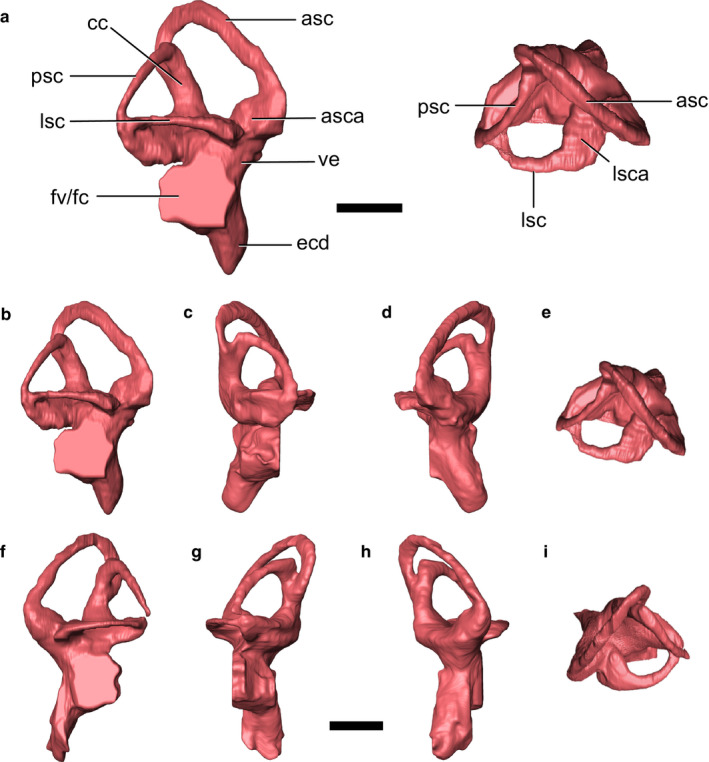
The endosseous labyrinth of IGM 100/976. (a) Labelled right labyrinth in lateral (left) and dorsal (right) views. The right (b–e) and left (f–i) labyrinths of IGM 100/976 shown in lateral (b,f), posterior (c,g), anterior (d,h), and dorsal (e,i) views. Scale bars: 5 mm. asc, anterior semicircular canal; asca, ampulla of the anterior semicircular canal; cc, crus communis; ecd, endosseous cochlear duct; fv/fc, fenestra vestibuli and fenestra cochleae (the division between the two cannot be identified); lsc, lateral semicircular canal; lsca, ampulla of the lateral semicircular canal; psc, posterior semicircular canal

Both endosseous labyrinths are preserved in IGM 100/976 (Figure 2), although the posterior portion of the left labyrinth, i.e. where the posterior semicircular canal meets the lateral canal, is not preserved (Figure 2f). In many respects, the vestibular anatomy of *V. mongoliensis* is similar to that of other non‐avian theropods (Balanoff *et al*., 2009; Witmer and Ridgely, 2009; Lautenschlager *et al*., 2012). Overall, the labyrinth has a somewhat triangular aspect in lateral view, with all semicircular canals being approximately orthogonal to each other. The anterior canal is taller than the posterior one and exhibits only a slight curvature until it curves sharply ventrally to help form the crus communis. The course of the anterior vertical canal is planar and has a roughly uniform lumen thickness along its entire length, except at the anterior ampulla where it meets the vestibule (Figure 2).

The posterior and lateral canals are approximately equal in length. The posterior canal deviates from planarity by exhibiting a slight sinusoidal curvature along its course. Although the posterior and lateral endosseous canals both appear to terminate posteriorly in a confluence (Figure 2), the posterior semicircular duct of the membranous labyrinth would have continued ventromedially and expanded into its component ampulla (as discussed in Neenan *et al*., 2018; Evers *et al*., 2019), and the lateral duct would have continued medially to meet the vestibule. Similar to palaeognath birds, but previously unknown in non‐avian theropods, the medial extremity of the posterior canal curves sharply ventrally and meets the crus communis in a position more anterolateral than the anterior canal (Carabajal and Succar, 2015; Benson *et al*., 2017).

The lateral canal emerges anteriorly from a large but dorsoventrally compressed ampulla. Its course is planar and highly curved in dorsal view, appearing to meet the vestibule at its posterior extreme just anterior to the posterior vertical canal (Figure 2a,e,i). As mentioned above, however, the membranous duct would have continued its loop medial to the posterior canal to meet the vestibule (e.g. Evers *et al*., 2019).

The cochlear duct, which would have housed the basilar papilla, the receptor organ for hearing, is relatively long in *V. mongoliensis* compared with most non‐avian theropods. It is also relatively wide and follows a similar anteriorly orientated course as the crus communis. The separation between the fenestra vestibuli and fenestra cochleae (oval and round windows, respectively) cannot be differentiated in this scan (Figure 2a,b,f).

## 4 DISCUSSION

### 4.1 Sensory abilities of *Velociraptor*

Floccular lobes are used to maintain head and eye stability during movement within vertebrates and, as such, are frequently linked to the agility of an organism (Witmer and Ridgely, 2009). As pointed out in Walsh *et al*. (2013) and Ferreira‐Cardoso *et al*. (2017), however, the size of the reconstructed flocculi do not necessarily reflect the actual volume of the lobes in life, as other anatomy (e.g. blood vessels) may have also resided within the floccular fossae, making them generally a poor indicator of flight style and ecology in birds (e.g. powered flight vs. gliding). Nevertheless, relatively large floccular fossae likely correlate with large flocculae despite extraneous anatomical structures, and Walsh *et al*. (2013) further postulate that enlarged flocculi in terrestrial birds could be an adaptation found in bipeds to help stabilize the unstable nature of bipedalism. It is therefore logical to interpret the floccular size in terrestrial, bipedal maniraptorans, such as dromaeosaurs, as relating to balance—with enlarged lobes corresponding to species that necessitated stable bipedal movement. The flocculi of IGM 100/976 are massive and suggest that quick movements and a stable gaze were essential to its everyday life (Figure 1). This interpretation fits well with the current idea that *V. mongoliensis* was a nimble predator that relied heavily on its agility while pursuing and attacking prey. Moreover, as enlarged floccular lobes have also been proposed to be indicative of strong vestibulo‐ocular (VOR) and vestibulocollic (VCR) reflexes (Hopson, 1977; Witmer and Ridgely, 2009), it can be reasoned that *V. mongoliensis* was able to track moving objects easily. With that being said, because the optic lobes were not preserved it is impossible to say at this point to what degree IGM 100/976 relied on sight rather than other senses. Based on the enlarged floccular lobes, elongated semicircular canals, and large orbit size of the species, it can be assumed that the visual acuity and field of view of *V. mongoliensis* was high (Stevens, 2006; Schmitz and Motani, 2011; Torres and Clarke, 2018). This heightened optical sensitivity is not surprising considering the hypothesized predatory lifestyle of *V. mongoliensis*. When combined with its large flocculi and potentially sensitive VOR and VCR, it is likely that *V. mongoliensis* was easily able to track and pursue its prey smoothly based on its sensory neuroanatomy and stereoscopic vision (as determined from the position of the orbits).

**FIGURE 3:**
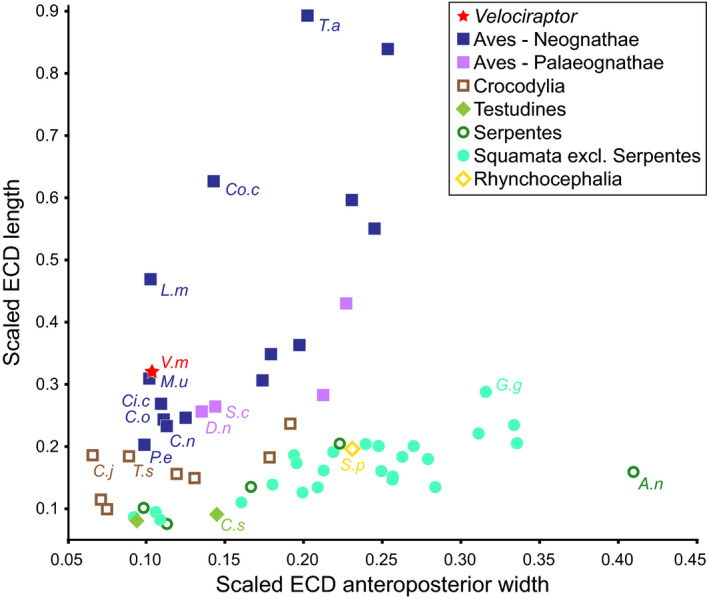
Scaled endosseous cochlear duct (ECD) length against scaled ECD anteroposterior width, with some taxa highlighted. *Velociraptor mongoliensis* grouped more closely with birds rather than wiith Crocodyliformes and non‐archosaurs. The scaled measurements of IGM 100/976 most closely resembled the upper range of vocal/social neognath birds. *A.n*, *Ahaetulla nasuta*; *C.j*, *Crocodylus johnstoni*; *C.n*, *Ciconia nigra*; *C.o*, *Cygnus olor*; *C.s*, *Chelydra serpentina*; *Ci.c*, *Ciconia ciconia*; *Co.c*, *Corvus corax*; *D.n*, *Dromaius novaehollandiae*; *G.g*, *Gymnodactylus geckoides*; *L.m*, *Luscinia megarhynchos*; *M.u*, *Melopsittacus undulatus*; *P.e*, *Psittacus erithacus*; *S.c*, *Struthio camelus*; *S.p*, *Sphenodon punctatus*; *T.a*, *Tyto alba*; *T.s*, *Tomistoma schlegelii*; *V.m*, *Velociraptor mongoliensis*. Modified from Walsh *et al*. (2009)

In life, the endosseous cochlear duct of *V. mongoliensis* would have housed the basilar papilla—the auditory organ of tetrapods (Gleich *et al*., 2005; Walsh *et al*., 2009). As the length of the cochlear duct has been interpreted as a rough measurement of the basilar papilla, the length of the duct can be used as an estimator of hearing frequencies in non‐avian dinosaurs (Witmer and Ridgely, 2009; Lautenschlager *et al*., 2012). Moreover, the relationship between the length of the cochlear duct and the basisphenoid has also been shown to correlate with hearing frequencies in modern archosaurs (Walsh *et al*., 2009), thus providing a way to calculate mean and high frequencies of non‐avian dinosaurs. A recent study using extant turkeys demonstrated that a shape analysis of a single endosseous labyrinth can be used to represent an entire population (Cerio and Witmer, 2019); we therefore suggest that the hearing frequencies calculated in this study can be used as proxies for high and average hearing frequencies for *V. mongoliensis*. By measuring and logarithmically transforming the ratio between the cochlear duct length and the total length of the basisphenoid, a mean hearing range (2,368 Hz) and high‐frequency hearing limit (3,965 Hz) was calculated for IGM 100/976—a range that is comparable to birds such as the common raven (*Corvus corax*) and the African penguin (*Spheniscus demersus*) (Walsh *et al*., 2009). Unsurprisingly, the scaled anteroposterior width and length of the cochlear duct were much more similar to birds—specifically neognaths such as budgerigars (*Melopsittacus undulatus*), storks (*Ciconia ciconia*), and mute swans (*Cygnus olor*) —than to more basal archosaurs and other reptiles (Figure 3).

Our results indicate that *V. mongoliensis* could hear, hunt, and perhaps vocalize most efficiently in the range of 2,400 Hz. When compared with other maniraptorans for which data are available, the mean hearing frequency of *V. mongoliensis* is high. For instance, the mean and high hearing frequencies estimated for the basally diverging therizinosaur *Falcarius utahensis* are 1,630 Hz and 4,000 Hz, respectively (Lautenschlager *et al*., 2012). Similarly, the frequency range of the more derived therizinosaurid *Erlikosaurus andrewsi* has a high range between 1,600 and 4,000 Hz (Lautenschlager *et al*., 2012). Although the mean frequency range of *V. mongoliensis* is notably higher than that of therizinosaurs, the high‐frequency values are roughly the same.

The elongate nature of the cochlear duct supports the hypothesis that *V. mongoliensis* was capable of detecting a wide range of sounds, indicating that hearing was likely an important sensory system in this taxon (Manley, 1990; Walsh *et al*., 2009; Witmer and Ridgely, 2009; Brusatte *et al*., 2016; Carabajal *et al*., 2016). In fact, IGM 100/976 plots closest to the social vocal learner *Melopsittacus undulatus* (budgerigar), making it feasible that *V. mongoliensis* utilized hearing in social interactions as well as active predation (Figure 3).

### 4.2 The trophic ecology of *Velociraptor*

Dinosaur feeding styles, such as predation vs. scavenging, have been a topic of popular interest in the past but remain difficult to diagnose in the fossil record (Holtz, 2008). Our current understanding of the trophic ecology of *V. mongoliensis* is provided by several sources. The most famous of these, the ‘fighting dinosaurs’ (IGM 100/25) of Mongolia, preserves what has been interpreted by some palaeontologists as a predation attempt of a *Velociraptor* on a *Protoceratops* (Carpenter, 1998). However, further evidence has emerged in recent years suggesting that *V. mongoliensis* was not an obligate predator. This includes *Velociraptor* tooth marks on bones, which have been interpreted as late stage scavenging, and the preserved gut contents of a subadult individual (Hone *et al*., 2010; 2012). Each of these specimens indicate that scavenging was a part of the trophic ecology of *V. mongoliensis*. The neuroanatomical results described in this study help flesh out the degree to which scavenging contributed to the diet of *V. mongoliensis*.

Our current understanding of the neuroanatomy of *V. mongoliensis* suggests that predation likely made up a large part of its diet. As with therizinosaurs and oviraptorosaurs, IGM 100/976 possesses relatively enlarged flocculi (Figure 1c,e; Lautenschlager *et al*., 2012; Balanoff *et al*., 2018). However, the flocculi of IGM 100/976 surpass those of observed therizinosaurs and almost completely fill the interior space between the semicircular canals. Enlarged flocculi have been used to predict prey tracking capabilities and may imply that the species had an acute vestibulo‐ocular reflex (Walsh *et al*., 2013). This evidence in conjunction with the wide field of binocular vision, extended hearing range (as determined from this study), and skeletal morphology indicates that rather than being equally or more reliant on scavenging, *V. mongoliensis* was well‐equipped to be an active predator.

The fossil record, however, indicates that scavenging was at least a small part of the diet in *V. mongoliensis* (Hone *et al*., 2010, 2012). Opportunistic scavenging is supported by gut contents recovered from MPC‐D100/54 that include a 75‐mm‐long bone of an unidentified pterosaur. Whether this represents an act of osteophagy or scavenging due to an injury or its small size, it is probable that the pterosaur was dead prior to being eaten, given its incomplete nature. In the case of IVPP V16137, a probable *Protoceratops*, multiple bone fragments including a dentary, exhibited bite marks characteristic of velociraptorine dromaeosaurs. Velociraptorine teeth (IVPP V16138) were also found in association with IVPP V16137, further indicating that a velociraptorine dromaeosaur was feeding on the carcass of IVPP V16137. Some of these tooth drag marks found along the anterior portion of the dentary suggest that this was an instance of late‐stage scavenging by *V. mongoliensis* due to the lack of significant muscle mass located along a dentary during life. While this evidence for scavenging can be interpreted as being somewhat circumstantial, we accept that the specimens nevertheless show enough evidence to be considered acts of scavenging rather than active predation based on the conclusions of previous studies (Hone *et al*., 2010; 2012).

This type of flexible hunting strategy is not surprising given that modern predator diets are a spectrum rather than an ‘either/or’ scenario in which seasonality, fitness, and other ecological constraints are the primary drivers (Mattisson *et al*., 2016). Here we propose the fossil evidence indicates a scavenging behaviour that complimented an active predatory lifestyle—similar to what can be found in the modern biota. Modern predators, including predatory birds such as *Aquila chrysaetos*, often resort to changes in hunting behaviour, or even scavenging, when prolonged weather patterns, injury or ontogenetic stage forces them to find alternative food sources (Tjernberg, 1981; Marchetti and Price, 1989; Wilmers *et al*., 2003; Mattisson *et al*., 2016). It follows that the neuroanatomy of *V. mongoliensis* suggests a behaviour that is adapted for active predation (Carpenter, 1998); however, young or injured individuals and those experiencing dietary constraints brought on by local climate would have actively sought out carcasses for an easy meal.

## 5 CONCLUSIONS

The neuroanatomy and sensory capabilities of *V. mongoliensis* are described here for the first time and are subsequently used to put this taxon into a larger palaeobiological context. Evidence from the hindbrain and labyrinth of IGM 100/976 suggests that *V. mongoliensis* had an average hearing range near 2,400 Hz (similar to modern social birds such as ravens and penguins), highly sensitive vestibulo‐ocular and vestibulocollic reflexes, and a fine‐tuned sense of balance—all of which would have been advantageous as an active predator. Although previous studies have used gut contents and tooth marks on *Protoceratops* mandibles to provide evidence that scavenging was a part of *V. mongoliensis* trophic ecology, most evidence, including the neuroanatomy, suggests an active predatory lifestyle. Therefore, we interpret the presence of scavenging as a facet of the trophic ecology for *V. mongoliensis*. Based on the behaviour of modern bird taxa, our better understanding of velociraptorine senses, the apparent case of a predation event in the ‘fighting dinosaurs’, and the age/health/environment of the scavenging individuals, it is likely that *V. mongoliensis* was an active predator that would readily rely on carrion in the event that a ready source of prey items was not available.

## CONFLICT OF INTEREST

The authors declare no conflict of interest.

## AUTHOR CONTRIBUTIONS

J.L.K. and J.M.N conceived the study. J.L.K reconstructed the 3D brain endocast, performed the sensory calculations, and drafted/revised the manuscript. J.M.N reconstructed and described the labyrinths and assisted in writing the manuscript. J.S.S. and J.A.G. provided the CT data, gave anatomical considerations for the project, and edited the initial drafts of the manuscript. A.M.B. provided expert knowledge and edited the final drafts of the paper.

## Data Availability

The 3D models produced in this study are openly available from MorphoSource at https://www.morphosource.org/Detail/ProjectDetail/Show/project_id/1019.
